# Preparation of a Novel Chitosan Based Biopolymer Dye and Application in Wood Dyeing

**DOI:** 10.3390/polym8090338

**Published:** 2016-09-10

**Authors:** Xiaoqian Wang, Ruilin Tang, Yang Zhang, Zhiming Yu, Chusheng Qi

**Affiliations:** 1 Beijing Key Laboratory of Wood Science and Engineering, Beijing Forestry University, Beijing 100083, China; xiaoqianwang1@126.com (X.W.); tangruilin@bjfu.edu.cn (R.T.); qichusheng@bjfu.edu.cn (C.Q.); 2 Ministry of Education Key Laboratory of Wooden Material Science and Application, Beijing Forestry University, Beijing 100083, China

**Keywords:** *O*-carboxymethyl chitosan, dye, preparation, antibacterial activity, water solubility, dyeing performance

## Abstract

A novel chitosan-based biopolymer dye possessing antibacterial properties was synthesized by reaction of *O*-carboxymethyl chitosan and Acid Red GR. The synthesized materials were characterized by Fourier transform infrared spectroscopy (FTIR), degree of substitution (DS), X-ray photoelectron spectroscopy (XPS), thermogravimetric analysis (TG), X-ray diffraction (XRD), water solubility test, antibacterial property test, and dyeing performance, including dye uptake, color difference, and fastness. Results showed that the synthesized dye was combined by –NH_3_^+^ of *O*-carboxymethyl chitosan and the sulfonic group of Acid Red GR. According to the comprehensive analysis of XRD and water solubility, the introduction of the carboxymethyl group and acid dye molecule changed the structure of the chitosan from compact to loose, which improved the synthesized dye’s water solubility. However, the thermal stability of the synthesized dye was decreased. The antibacterial property of the poplar wood dyed with the synthesized dye was enhanced and its antibacterial rate, specifically against *Staphylococcus aureus* and *Escherichia coli*, also increased to a rate of more than 99%. However, the dye uptake of the synthesized dye was lower than that of the original dye. Despite this, though, the dyeing effect of the synthesized dye demonstrated better water-fastness, and light-fastness than the original dye. Therefore, the novel chitosan-based biopolymer dye can be a promising product for wood dyeing.

## 1. Introduction

Chitosan is the second most abundant natural biopolymer after cellulose and is a cationic polysaccharide, which is extracted from chitin by deacetylation and is formed primarily of the repeating units of β-(1,4)-2-amino-2-deoxy-d-glucose (or d-glucosamine) [1,2,3,4]. It has special chemical and physical properties, such as biodegradability, biocompatibility, renewability, antibacterial activity, and nontoxicity [5,6,7,8,9]. And due to these excellent properties, chitosan has been widely applied in waste water treatments of heavy metals and dyes, as an ion adsorbent, in biomedicine, as a drug and gene carrier, in finishing textiles, as an antibacterial agent, and so on [10,11,12,13,14,15,16]. However, chitosan has low solubility in water due to its inter-and intra-molecular structure of hydrogen bonds, which limits its applications in the wood, food, and other industries [17].

To improve the water solubility of chitosan, considerable studies have been conducted to make various water-soluble chitosan derivatives, through chemical modification [18,19,20]. Among these derivatives are carboxymethyl chitosan, which has better solubility and is used in this study [21,22,23].

So far, antibacterial dyes, including naturally-extracted dyes and chemical synthetic dyes, have been the subject of intense research [24]. Naturally-extracted dyes have a lower toxicity than synthetic dyes, but the available colors are more limited [25]. The chemical modification of dyes, with functional groups, is an efficient and inexpensive way to obtain multicolored antibacterial dyes. Furthermore, color is a principal aspect in the wood industry for determining the degree of appreciation by consumers [26]. Acid dye is widely used in wood dyeing for its bright colors, complete color spectrum and the manner in which it easily penetrates wood in an acidic environment. However, acid dye’s molecular weight is very small, which means the acid dye does not absorb well into the wood and runs off easily [27]. In addition, the decolorization of dye is seriously harmful to human health. Therefore, the novel chitosan-based biopolymer dye, which is prepared by acid dye and anti-bacterial chitosan, would have a potential market in the wood industry for special situations, such as hospitals, etc.

In this work, we employed an approach to synthesize and test the properties of the novel chitosan based biopolymer dye. The chemical structures, crystallinity, atom percentage, and thermal stability of chitosan based biopolymer dye were detected and analyzed by Fourier transform infrared spectroscopy (FTIR), X-ray diffraction (XRD), X-ray photoelectron spectroscopy (XPS), and thermogravimetric analysis (TG). In addition to this, we evaluated the antibacterial activity of the novel chitosan based biopolymer dye against the *Staphylococcus aureus* (*S. aureus*) and *Escherichia coli* (*E. coli*) with JIS Z 2801:2000 control. The water solubility of the novel chitosan based biopolymer dye was examined by UV–visible spectroscopy (UV–VIS). We also tested dyeing properties, including dye uptake, color difference, and color fastness.

## 2. Materials and Methods

### 2.1. Materials

Chitosan, which has, when measured by viscometry, a molecular weight of 4.5 × 10^5^ and an 85% degree of deacetylation, was supplied by Sinopharm Chemical Reagent Co., Ltd. (Shanghai, China). Monochloroacetic acid and isopropyl alcohol were purchased from Tianjin Chemical Reagent Co., Tianjin, China. The Acid Red GR (*C.I. Acid Red 73*) was provided by the Taopu Dye Plant in Shanghai, China. Poplar wood veneers (50 mm × 50 mm × 0.8 mm), with 8% moisture content, were obtained from Harbin, China. All other agents were of analytical grade.

### 2.2. Preparation of Chitosan-Based Biopolymer Dye

#### 2.2.1. Preparation of *O*-Carboxymethyl Chitosan (*O*-CMCS)

Firstly, *O*-CMCS was prepared according to the previous report, with some modifications [28]. Chitosan (10.0 g), NaOH (13.5 g), and isopropyl alcohol (150 mL) were added into a flask (250 mL) to swell and alkalize at −20 °C for 24 h. Next, monochloroacetic acid (15.0 g) was dissolved in isopropyl alcohol (20 mL). Then, the mixed solution was added into the reaction system drop-wise at regular intervals for 30 min, and the reaction was kept at 50 °C for 6 h with continuous stirring. After this, the reaction was completed by adding ethyl alcohol (70%, 400 mL). The reaction products were then filtered and first washed five times, by ethyl alcohol (70%), and then washed by absolute alcohol to remove any remaining salt and water. The primary obtained product was *O*-carboxymethyl chitosan sodium salt. Then, the produced *O*-carboxymethyl chitosan sodium salt was then immersed into aqueous ethyl alcohol (80%, 200 mL) after which hydrochloric acid was added (35%–37%, 15 mL). After stirring for 30 min, the filtration residue was collected and washed by ethyl alcohol (80%) to neutralize it. The *O*-CMCS of the H-form [29] of the resulting products was vacuum dried for 48 h. The point of zero charge (pH_PZC_) of the samples was determined by the solid addition method [30].

#### 2.2.2. Preparation of Chitosan-Based Biopolymer Dye

**Figure 1:**
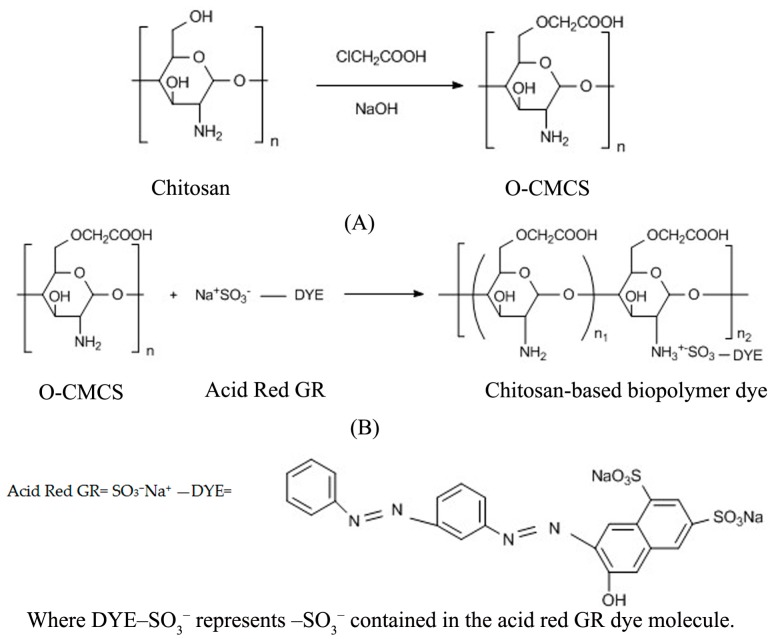
Chemical reaction equations of (**A**) *O*-CMCS and (**B**) chitosan-based biopolymer dye.

*O*-CMCS (5.0 g) was dissolved into 300 mL of deionized water and stirred until completely dissolved. After that, the Acid Red GR solution (20 g/L, 15 mL) was added into the flask, drop by drop, and reacted for 5 h with continuous stirring at 60 °C. The reaction products were then filtered to obtain a residue. The residue was rinsed with ethyl alcohol (90%, 300 mL) and absolute alcohol until the filtrate was colorless, and then dried in vacuo at 40 °C for 12 h. The dried product was ground thoroughly into a power, and stored for characterization. The chemical reaction equation of the chitosan based biopolymer dye is shown in Figure 1 [31,32]. In this chemical equation, the symbolic chemical formula DYE–SO_3_^−^ means that –SO_3_^−^ group is contained in the dye molecule. According to the stereo-hindrance effect and molecular structure, most of chitosan biopolymer dye molecules have only one reacted –SO_3_^−^ group per molecule, and there can be two reacted –SO_3_^−^ groups on one dye molecule [27].

### 2.3. Characterization

#### 2.3.1. Fourier Transform Infrared Spectroscopy (FTIR) Analysis

The FTIR characterization of the chitosan, *O*-CMCS, and the chitosan-based biopolymer dye was carried out using a FTIR spectrophotometer (Nicolet 6700 Thermo Scientific, Exton, PA, USA). The samples were analyzed in the range of 4000–400 cm^−1^ at a resolution of 4 cm^−1^ for 32 scans.

#### 2.3.2. Degree of Substitution (DS) of *O*-CMCS

The degree of substitution (DS) of *O*-CMCS was measured using the potentiometric titration method and the degree of reaction of the chitosan based biopolymer dye was calculated by element analysis of XPS. The *O*-CMCS (0.3 g) was dissolved in HCl standard solution (0.1 M). The above solution was then titrated with NaOH standard solution (0.1 M) and the pH value of the solution was simultaneously recorded. The second-order derivative method was used to obtain the sudden-change point. The DS can be calculated as follows [33]:

(1) $$A=\;\frac{\left(V_{2}\;-\;V_{1}\right)C}{m};\;\mathrm{DS}=\frac{0.203A}{1\;-\;0.058A}$$

where *V*_1_ is the potentiometric titration end-point of excessive HCl (mL); *V*_2_ is the potentiometric titration end-point of –COOH (mL); C is the molar concentration of NaOH solution (mol/L); and m is the weight of *O*-CMCS (g).

#### 2.3.3. X-Ray Photoelectron Spectroscopy (XPS) Analysis

To confirm the composition of the chitosan, *O*-CMCS, and the chitosan-based biopolymer dye, XPS (K-Alpha Thermo Scientific, Exton, PA, USA) was used to analyze the materials with a monochromatic Al Kα X-ray source at a pressure of 10^−8^ mbar. Survey data were recorded with 1.0-eV steps and 200-eV analyzer pass energy, and high-resolution regions were analyzed and recorded with 0.1-eV steps and 50-eV pass energy. The binding energies were calibrated with the C1s peak at 285 eV as a reference.

#### 2.3.4. X-Ray Diffraction (XRD) Analysis

The crystallinity of the chitosan, *O*-CMCS, and the chitosan-based biopolymer dye was evaluated by the XRD method using an XRD 6000 diffractrometer (Shimadzu, Kyoto, Japan) with Cu Kα radiation with a graphite monochromator. The diffraction data were collected 2θ from 10° to 80° in a fixed time mode with a step interval of 0.02°.

#### 2.3.5. Thermogravimetric (TG) Analysis

The thermal properties of the chitosan, *O*-CMCS, and the chitosan-based biopolymer dye were measured with a Q5000 TG analyzer (TG Instruments, Austin, TX, USA). The samples were heated from room temperature to 700 °C at a constant heating rate of 10 °C/min in a flowing nitrogen atmosphere.

#### 2.3.6. Antibacterial Activity Study

The antibacterial activity of the chitosan-based biopolymer dye, which was used to dye poplar veneers, was determined according to JIS Z 2801:2000. The test method was as follows: (1) the poplar wood veneers were cut into 50 mm × 50 mm × 0.8 mm (length × width × thickness) and used as the standard test size pieces. Six pieces of the untreated veneers and three pieces of the antimicrobial veneers were prepared; (2) in the process of inoculation of the test inoculums, each test piece was placed in a sterilized Petri dish, upon which the test surface consisted of. 0.4 mL of the *E. coli* (ATCC 1229) inoculum exactly taken with a pipette and instilled onto each test piece in the Petri dish. The test inoculum was then covered with a film, and the film was pressed so that the test inoculum spread over the film; (3) the Petri dish containing the test pieces, which was inoculated with the test inoculums (three untreated test pieces and three antimicrobial test pieces) was incubated at a temperature of 35 ± 1 °C and a relative humidity of not less than 90% for 24 ± 1 h; and (4) the antimicrobial efficacy of the product is evaluated using the value of antimicrobial activity data obtained from the test at the incubation temperature specified. The test method of *S. aureus* (ATCC 6538) was the same as the test method of *E. coli* (ATCC 1229).

#### 2.3.7. Water Solubility Test

Water solubility of the chitosan-based biopolymer dye was determined by UV–VIS spectroscopy (721 ApL, Shanghai, China) [34]. The water solubility of the chitosan-based biopolymer dye (*w*/*v* = 50 mg/20 mL) was measured in the pH values of 2, 3, 4, 5, 6, and 7 at λ_max_ of 510 nm. Meanwhile, 0.25 wt % of Acid Red GR solution, which had a concentration equal to the chitosan-based biopolymer dye, was used as reference.

#### 2.3.8. Color Difference and Color Fastness Test

An atmospheric pressure dip-dye method was used for wood veneer dyeing. All of the experimental groups were heated to a temperature of 80 °C and a dipping time of 180 min in an electrothermostatic water bath. The dosage of the Acid Red GR and chitosan-based biopolymer dyes used in poplar veneers dyeing were in the same molecular proportion of dye (0.005 mol/L, 300 mL). The color difference between the poplar veneers dyed with the chitosan-based biopolymer dye and the Acid Red GR was determined with a color-measuring instrument (Dataflash 110 Datacolor, Austin, TX, USA) according to CIELAB color coordinates. L^*^ (lightness and darkness), a^*^ (redness and greenness), b^*^ (yellowness and blueness) were measured. The difference in the color parameters ΔL^*^, Δa^*^, Δb^*^, and ΔE^*^ (total color difference) were measured at five positions in each veneer [35].

(2) $$\Delta L^{*}\;=\;L_{1}^{*}-L_{0}^{*}$$

(3) $$\Delta a^{*}\;=\;a_{1}^{*}\;-\;a_{0}^{*}$$

(4) $$\Delta b^{*}=\;b_{1}^{*}-\;b_{0}^{*}$$

(5) $$\Delta E^{*}={\left(\;\Delta L^{*2}+\;\Delta a^{*2}+\;\Delta b^{*2}\right)}^{1/2}$$

To determine the light fastness, half of each groups’ veneers were exposed to YG611, a light and weather fastness tester (Changzhou No.2 Textile Instrument Factory, Changzhou, China) under a xenon lamp for 2 h, continuously. Other veneers were maintained in 80 °C water for 2 h. The color differences of the specimens before ($L_{0}^{*}$, $a_{0}^{*}$, $b_{0}^{*}$) and after ($L_{1}^{*}$, $a_{1}^{*}$, $b_{1}^{*}$) treatments were calculated with Equations (2)–(4), and ΔE^*^ was calculated with Equation (5).

#### 2.3.9. Measurement of Dye Uptake

The amount of dye pickup by the wood veneer during dyeing was measured to determine the absorption of the dyes. A UV–visible spectroscopy (721 ApL, Shanghai, China), which found the maximum absorption wavelength (λ_max_) for the novel dye and original dye to be 510 nm, was used to determine the absorption. Percent exhaustion (*C*_t_%) of the dye solution was calculated based on the following equation [36]:

(6) $$Ct\;\left(\%\right)\;=\frac{A_{0-}A_{t}}{A_{0}}\;\times 100$$

where *A*_0_ and *A*_t_ represent the absorption of the dye solution at λ_max_ separately before and after the wood veneer dyeing, respectively.

## 3. Results and Discussion

### 3.1. FTIR Analysis

**Figure 2:**
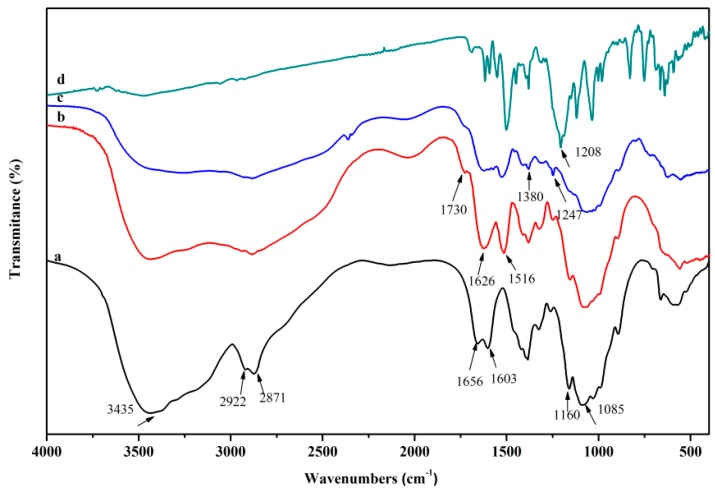
FTIR spectra of a, chitosan; b, *O*-CMCS; c, chitosan-based biopolymer dye; and d, Acid Red GR.

The FTIR spectra of chitosan, *O*-CMCS, chitosan-based biopolymer dye, and Acid Red GR are shown in Figure 2. The basic characteristic peaks of the chitosan were shown at: 3435 cm^−1^ (O–H stretch and N–H stretch, overlapped), 2922 and 2871 cm^−1^ (C–H stretch), 1656 cm^−1^ (NH_2_ deformation), 1603 cm^−1^ (N–H bend), 1160 cm^−1^ (bridge –O– stretch), 1085 cm^−1^ (C–O stretch, secondary hydroxyl group), and 1030 cm^−1^ (C–O stretch, primary hydroxyl group), as reported in the literature [37,38]. Compared with the spectrum of the chitosan, the new absorption peak at 1730 cm^−1^ belonged to the –COOH stretch, illustrating the success of the introduction of a carboxymethyl group [39]. Moreover, the peak associated with the primary hydroxyl group (1030 cm^−1^) in the spectrum of chitosan disappeared in the spectrum of *O*-CMCS, whilst the absorption peak of the secondary hydroxyl group (1085 cm^−1^) did not change, which demonstrates that the carboxymethylation primarily occurred at the C6–OH of the chitosan [40]. In addition to this, the peaks at 1626 cm^−1^ and 1516 cm^−1^ correspond to the anti-symmetric stretch and symmetric stretch of the –NH_3_^+^ groups of *O*-CMCS [41]. In the spectrum of chitosan-based biopolymer dye, the peak at 1380 cm^−1^ is the typical peak of C–H vibration of the benzene ring in the Acid Red GR dye [27]. This also appeared in the spectrum of the chitosan-based biopolymer dye after modifications. This demonstrated that some Acid Red GR dye molecules were combined with *O*-CMCS. The sulfonate group (1208 cm^−1^) of acid dye bonded with the amino group of *O*-CMCS under acidic conditions [42], and the sulfonate group was attracted to the –NH_3_^+^ in the chitosan-based biopolymer dye, so it was shown at 1247 cm^−1^ in the chitosan-based biopolymer dye.

### 3.2. DS of O-CMCS Analysis

**Figure 3:**
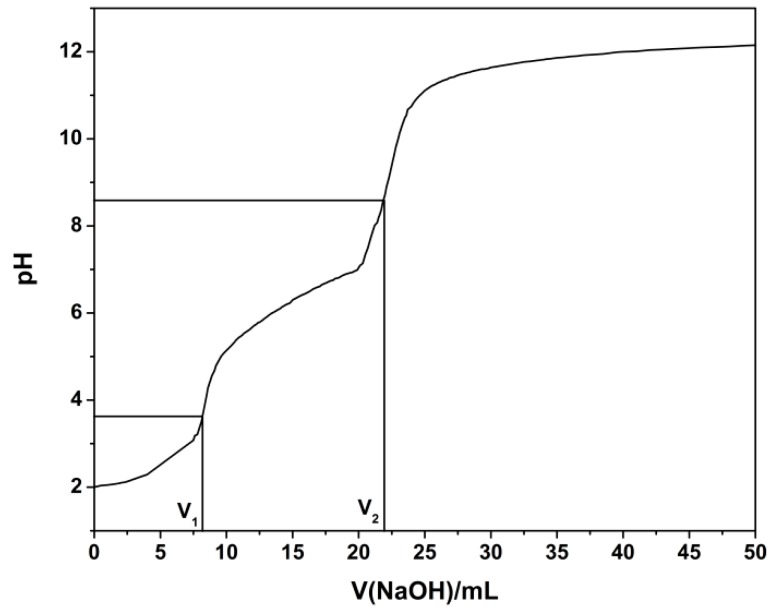
Potentiometric titration curve of *O*-CMCS.

The potentiometric titration curve, as is shown in Figure 3, was drawn based on the pH value and the volume of NaOH solution. The DS of *O*-CMCS was 1.27, illustrating that the carboxymethyl group was introduced onto OH at C6 and C3. Furthermore, the steric hindrance of OH at C6 is obviously far smaller than that of OH at C3, thus showing carboxymethylation mainly occurred at C6 [33]. The results concurred with the results of the FTIR.

### 3.3. XPS Analysis

**Figure 4:**
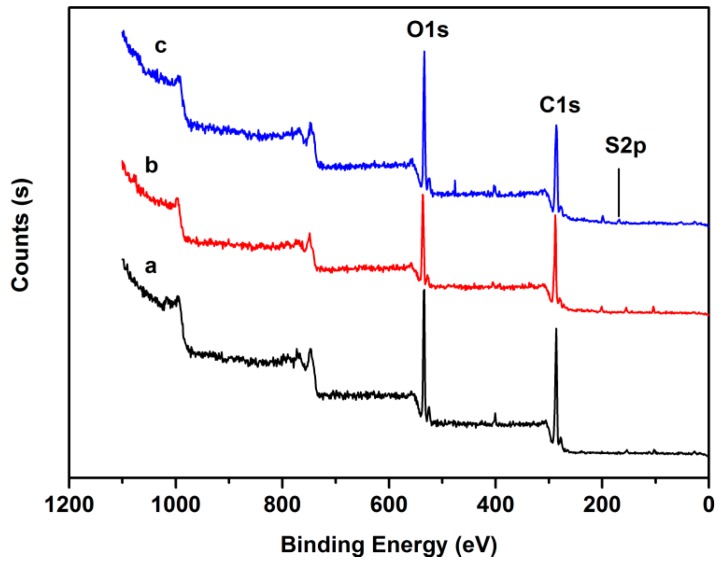
XPS spectra of a, chitosan; b, *O*-CMCS; and c, chitosan-based biopolymer dye.

Figure 4 shows the typical XPS survey spectra for the chitosan, *O*-CMCS, and the chitosan-based biopolymer dye. For chitosan, the absolute differences in mol % were 71.38 and 23.21 at % (atom percentage) for C and O, respectively. Whilst the absolute differences of *O*-CMCS were 70.72 and 26.06 at % for C and O, respectively. These results illustrated that the carboxymethyl group was introduced onto OH at C6 and C3. For the chitosan based biopolymer dye, the absolute differences were 1.31 and 6.83 at % for S and N, respectively. The S element, which was contained in the Acid Red GR dye, was detected in the synthetic dye, which proves the Acid Red GR dye molecule was combined with the *O*-CMCS. All of the above results further proved that the chitosan-based biopolymer dye was successfully prepared. Through the atom percentage analysis, almost 15.56% of the *O*-CMCS units reacted with the dye molecules and this was effective enough for the dyeing effect, water solubility, and antibacterial activity of the chitosan-based biopolymer dye.

### 3.4. XRD and Water Solubility Analysis

**Figure 5:**
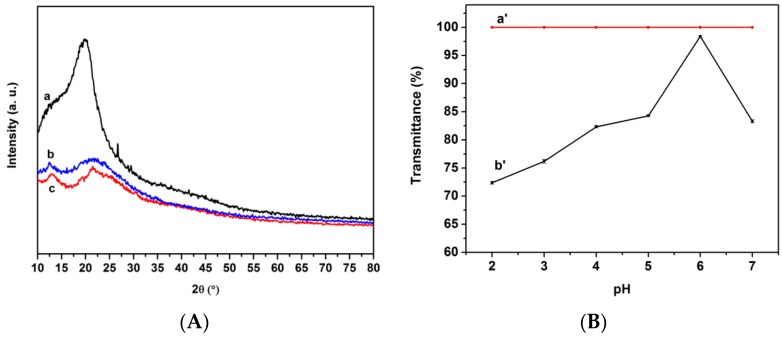
(**A**) XRD patterns of a, chitosan, b, *O*-CMCS, c, chitosan-based biopolymer dye; (**B**) water solubility of a’, Acid Red GR dye; b’, chitosan-based biopolymer dye at different pH values.

The XRD patterns of the chitosan, *O*-CMCS, the chitosan-based biopolymer dye, as well as the water solubility of the Acid Red GR dye and the chitosan-based biopolymer dye at different pH values ranging from 2 to 7 are shown in Figure 5. The X-ray diffraction pattern of the chitosan showed a characteristic peak at 2θ = 20.0°, though it decreased in the *O*-CMCS. The introduction of the carboxymethyl group had a great effect on the crystal structure of the *O*-CMCS. The decreasing intensity of the diffraction peak of *O*-CMCS demonstrates that it has a looser structure than the chitosan due to the disruption of bonding between the chitosan molecules. The XRD results also revealed that crystalline regions almost disappeared due to the introduction of sulfonate group of Acid Red GR. It showed that inter- and intra-molecular hydrogen bonds of *O*-CMCS had decreased after being combined with the Acid Red GR dye. Thus, the solubility of the chitosan-based biopolymer dye was increased. However, the water solubility of the mixture of dye and chitosan cannot be detected with UV–VIS for forming a lot of precipitation. As can be seen from Figure 5, the water solubility of the chitosan based biopolymer dye was increased with the increasing pH values from 2 to 6 and then slightly decreased. In this study, pH_PZC_ for *O*-CMCS was 6.3. Usually, as an amphoteric polyelectrolyte, *O*-CMCS has a different structure in acidic and alkaline media [43]. When the pH of the chitosan-based biopolymer dye was higher than 6.3, the –NH_3_^+^ could be converted to –NH_2_. This resulted in partial decomposition of the synthetic dye, which caused the water solubility to decrease. The transmittance of the chitosan-based biopolymer dye was higher than 70%. Furthermore, when the pH value was 6, the transmittance was higher than 90% and was recorded at 98.4%, specifically. These results illustrated that the chitosan based biopolymer dye had excellent water solubility in acidic conditions.

### 3.5. TG Analysis

**Figure 6:**
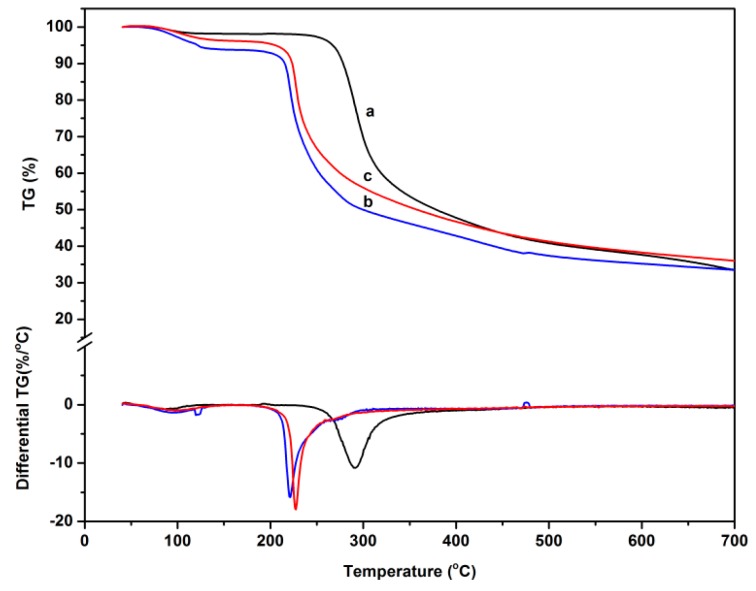
TG and DTG curves of a, chitosan; b, *O*-CMCS; and c, chitosan-based biopolymer dye.

The thermal stability of the chitosan, *O*-CMCS, and the chitosan-based biopolymer dye was investigated using TG. Weight loss traces and their differential TG curves, which were recorded within room temperature to 700 °C, are shown in Figure 6, and the thermogravimetric parameters for the samples are presented in Table 1. For the chitosan, there were two stages in the degradation. The first stage started slowly at 85 °C, which was associated with the release of the water molecules. The second stage was observed at 245 °C. This stage was related to the decomposition of the chitosan backbone [44]. The maximum rate of weight loss of the chitosan was shown with a weight loss of 48% between 245 and 390 °C. In addition to this, the residue was 33.5%. For *O*-CMCS, there were two distinct weight loss stages in the degradation. The onset degradation temperature (*T*_ei_) was 49 °C, which demonstrated that the thermal stability of the chitosan was higher than that of the *O*-CMCS. Moreover, the maximum rate of weight loss of the *O*-CMCS was 43% between 205 and 280 °C. The char residue (30.5%) was smaller than that of the chitosan. These results illustrated that the intramolecular hydrogen bonding structure of the chitosan decreased due to the introduction of the carboxymethyl group. For the chitosan-based biopolymer dye, the *T*_ei_ was 46 °C. The further introduction of dye molecules to the *O*-CMCS caused the inter-and intra-molecular hydrogen bonds to weaken, thus, the thermal stability of the novel dye was the worst of the three samples. In addition to this, the maximum rate of weight loss of the novel dye was 45% between 205 and 280 °C. The char residue was 34.1%, a result that may be due to the fact that the structure of the Acid Red GR, classified as a diazo dye, is a stable ‘trans’ form, which contributes to a higher carbon residue.

### 3.6. Antibacterial Activity Study

The antibacterial activity of dyed veneers with Acid Red GR and dyed veneers with chitosan-based biopolymer dye was investigated against *S. aureus* and *E. coil*, the results of which are provided in Table 2. Compared with the reduction of the veneers that were dyed with Acid Red GR, the reduction of the dyed veneers with chitosan based biopolymer dye was increased from 72% to more than 99% against *S. aureus* and from 78% to more than 99% against *E. coil*. This result was attributed to the fact that in an acidic solution –NH_2_ of *O*-CMCS was transformed into the antibacterial group –NH_3_^+^, and some of –NH_3_^+^, which did not react with the Acid Red GR, was responsible for the antibacterial activity [33]. Moreover, the interaction between the positive-charge chitosan derivatives and the negative-charge cell envelope was improved [27]. Hence, the chitosan-based biopolymer dye possessed positive antibacterial properties.

### 3.7. Color Difference and Color Fastness Test

The relationship between the color of the poplar veneers dyed with Acid Red GR and chitosan-based biopolymer dye is shown in Table 3. ΔE^*^ was 4.82 ± 0.62, indicating that the chitosan-based biopolymer dye could change the dyeing effect slightly. a^*^ improved a little, which illustrates that the chitosan-based biopolymer dye mainly changed the color of redness and greenness.

The color fastness of the poplar veneers dyed with chitosan based biopolymer dye was remarkably enhanced according to Table 4. △E^*^ decreased from 13.96 ± 0.40 to 10.23 ± 0.42 for water-fastness and decreased from 13.29 ± 0.99 to 7.43 ± 0.63 for light-fastness. These results can be attributed to the fact that the membrane consisted of chitosan-based biopolymer dye can protect Acid Red GR dye molecules from running off and prevent photooxide and photofade reactions [45]. In addition, any dye molecules that are exposed to the air would change the color [46]. The molecular volume of the chitosan-based biopolymer dye was also improved, which means the number of combined dye molecules was decreased compared with Acid Red GR dye in the same area of veneer. Thus, overall, the color fastness of veneers dyed with the chitosan-based biopolymer dye was improved.

### 3.8. Dye Uptake Analysis

**Figure 7:**
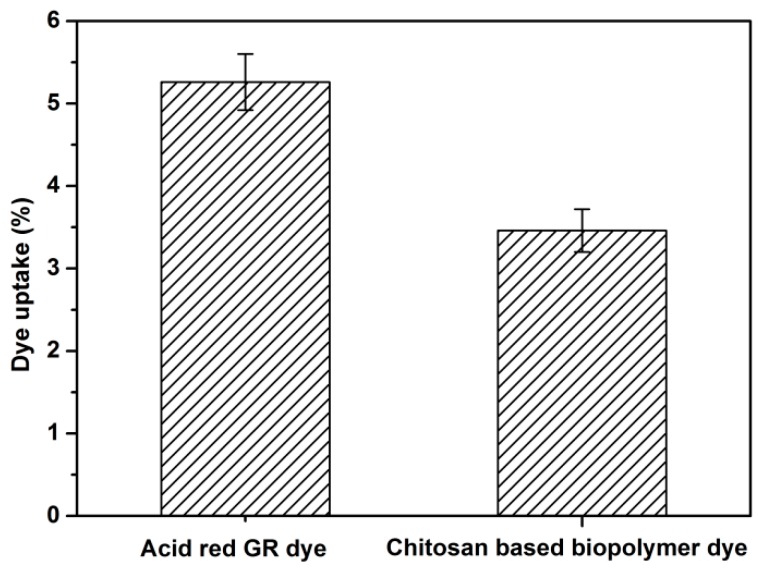
Dye uptake of Acid Red GR and chitosan-based biopolymer dye.

Figure 7 shows the dye uptake of the Acid Red GR and the chitosan-based biopolymer dye. The dye uptake for the Acid Red GR was 5.26%, while the dye uptake for the chitosan-based biopolymer dye (3.46%) was lower than that of the original dye. The reason for this may be due to the fact that the molecular weight of the chitosan-based biopolymer dye was increased after chemical modification. Hence, the number of dye molecules was decreased compared with Acid Red GR dye in the same area of veneer when dyeing wood veneers. In addition, the viscosity of the novel dye hindered the diffusion and penetration of dye molecules into the wood fibers. Still, the color fastness and antibacterial activity of wood veneers dyed with the novel dye were both improved. Thus, it is most beneficial for dyeing wood with the novel dye, and this is especially true for hospitals, outdoor decoration, and so on.

## 4. Conclusions

The novel chitosan-based biopolymer dye was successfully prepared through the combination of the sulfonate group of Acid Red GR dye and –NH_3_^+^ of *O*-CMCS in an acidic environment. The novel chitosan based biopolymer dye had excellent antibacterial activity against *S.* *aureus* and *E. coli* and the antibacterial rates were both more than 99%. Water solubility also improved and expressed the highest levels when the pH value was 6. Moreover, the novel chitosan-based biopolymer dye was proved to have a better water-fastness and light-fastness and changed the dyeing effect slightly. The novel chitosan-based biopolymer dye can be promising for wood dyeing.

## Figures and Tables

**Table 1:** Thermogravimetric parameters for thermal degradation of chitosan, *O*-CMCS, and chitosan-based biopolymer dye.

Sample	*T*_ei_ (°C)	*T*_max1_ (°C)	*T*_max2_ (°C)	Char Residue at 700 °C (%)
Chitosan	85	88	291	33.5
*O*-CMCS	49	95	221	30.5
Chitosan-based biopolymer dye	46	101	233	34.1

**Table 2:** Antibacterial activity of chitosan-based biopolymer dye.

Sample	*S. aureus*			*E. coil*		
	Concentration of Bacteria (CFU/cm^2^)		Reduction (%)	Concentration of Bacteria (CFU/cm^2^)		Reduction (%)
	0 h	24 h		0 h	24 h	
Blank control	1.8 × 10^4^	1.8 × 10^6^	–	2.3 × 10^4^	1.7 × 10^6^	–
Dyed poplar wood with Acid Red GR	1.7 × 10^4^	4.2 × 10^3^	72	2.2 × 10^4^	4.1 × 10^3^	78
Dyed poplar wood with chitosan based biopolymer dye	–	<1.3	>99	–	<1.3	>99

**Table 3:** Color differences of poplar veneers dyed with Acid Red GR and chitosan-based biopolymer dye.

Color Parameters	Dye	Chitosan-Based Biopolymer Dye	Magnitude ^1^
L^*^	70.98 ± 1.87	68.96 ± 1.17	−2.03 ± 2.04
a^*^	19.37 ± 2.19	22.73 ± 2.01	3.36 ± 1.95
b^*^	19.50 ± 1.26	20.35 ± 0.81	0.86 ± 0.99
ΔE^*^^2^	–	4.82 ± 0.62	–

^1^ Magnitude was calculated with Equations (2), (3), and (4); ^2^ ΔE^*^ was calculated with Equation (5).

**Table 4:** Color fastness of poplar veneers dyed with Acid Red GR and chitosan-based biopolymer dye.

Color Parameter ^1^	Water-Fastness		Light-Fastness	
	Dye	Chitosan-Based Biopolymer Dye	Dye	Chitosan-Based Biopolymer Dye
ΔL^*^	5.36 ± 0.34	2.10 ± 0.79	−8.45 ± 1.17	−1.98 ± 0.40
Δa^*^	−12.53 ± 0.35	−8.38 ± 0.19	−10.20 ± 0.40	−3.62 ± 0.09
Δb^*^	−3.03 ± 0.24	−5.44 ± 0.45	−0.14 ± 1.08	6.15 ± 0.74
ΔE^*^^2^	13.96 ± 0.40	10.23 ± 0.42	13.29 ± 0.99	7.43 ± 0.63

^1^ The color parameter was calculated using Equations (2)–(4); ^2^ ΔE^*^ was calculated with Equation (5).
